# Pesticide exposure and risk of bladder cancer: A meta-analysis

**DOI:** 10.18632/oncotarget.11397

**Published:** 2016-08-19

**Authors:** Zhen Liang, Xiao Wang, Bo Xie, Yi Zhu, Jian Wu, Shiqi Li, Shuai Meng, Xiangyi Zheng, Alin Ji, Liping Xie

**Affiliations:** ^1^ Department of Urology, The First Affiliated Hospital, School of Medicine, Zhejiang University, Hangzhou, Zhejiang Province, People's Republic of China; ^2^ Department of Urology, Tongde Hospital of Zhejiang Province, Hangzhou, Zhejiang Province, People's Republic of China; ^3^ Department of Urology, Zhejiang Provincial People's Hospital, Hangzhou, Zhejiang Province, People's Republic of China

**Keywords:** pesticide exposure, bladder cancer, meta-analysis, epidemiology

## Abstract

**Objective:**

We conducted a meta-analysis to quantitatively evaluate the correlation between pesticide exposure and the risk of bladder cancer by summarizing the results of published case-control and cohort studies.

**Methods:**

A systematic literature search of articles update to February 2015 was conducted via Pubmed, Web of Science, Cochrane Library, and the Chinese National Knowledge Infrastructure (CNKI) databases, and the references of the retrieved articles. Fixed- or random-effect models were used to summarize the estimates of OR with 95% CIs for the highest versus the lowest exposure of pesticide.

**Results:**

The pooled OR estimates indicated that pesticide exposure was associated with an increased risk of bladder cancer (OR=1.649, 95% CI 1.223-2.223). In subgroup analysis, we detected pesticide exposure demonstrated as a significant risk factor on bladder cancer in America (OR=1.741, 95% CI 1.270-2.388). Similar results were discovered in both case-control group and cohort group (OR=2.075, 95% CI 1.183-3.638, OR=1.146, 95% CI 1.074-1.223, respectively). No evidence of publication bias was found by Begg's or Egger's test (P = 0.210, P = 0.358, respectively).

**Conclusion:**

In conclusion, our meta-analysis indicated that pesticide exposure was associated with an increased risk of bladder cancer. Further researches should be conducted to confirm the findings in our study and better clarify the potential biological mechanisms.

## INTRODUCTION

Bladder cancer is generally accepted as the 11th most commonly diagnosed type of cancer, and the incidence of bladder cancer is reported to be elevating worldwide [1]. In the United States, statistics demonstrated that an estimated 74,690 cases were newly diagnosed bladder cancer, among which 15,580 were expected to die in 2014 [2]. Bladder cancer has become a serious social problem due to its elevating incidence and recurrence rate. It is suggested that both environmental and genetic factors play critical roles in the development of bladder cancer [3–5]. However, the exact mechanisms are still not well elucidated. Therefore, understanding the potential carcinogenetic interaction between environmental and genetic factors is important to identify potential risk factors of bladder cancer.

It was reported that both environmental and occupational exposures could be potential causes of several types of cancer [5]. Therefore, many epidemiologic researches were carried out to evaluate the relationship between the risk of bladder cancer and several occupational exposures [5]. Pesticide use has increased over 50% and its toxicity has elevated ten-fold since 1950 [6]. Pesticide exposure is considered to be associated with increasing cancer risk via resulting in chromosomal aberrations, oxidative stress or cell signaling disturbances [7–9]. Nevertheless, the findings on the correlation between pesticide exposure and the risk of bladder cancer are inconsistent.

Meta-analysis is considered to be a valuable tool for demonstrating trends, which might not be apparent in a single study. Therefore, summarizing independent studies increase the confidence in the results [10]. To the best of our knowledge, no meta-analysis regarding the correlation between pesticide exposure and the risk of bladder cancer has been published before. The purpose of the present study was to quantitatively evaluate the correlation between pesticide exposure and the risk of bladder cancer by summarizing the results of published case–control and cohort studies.

## RESULTS

### Description of the meta-analysis

**Figure 1:**
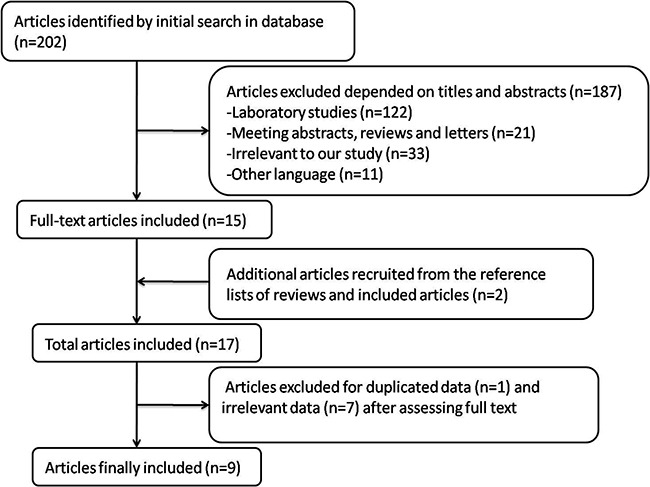
Process of article selection

A total of 202 articles were identified when “(Bladder cancer) AND (pesticides OR herbicides OR fungicides OR insecticides) were used as keywords for article searching. After a closer screening, 187 articles were excluded according to titles and abstracts. 1 article was excluded due to duplicated data after assessing the full text [11], and 7 articles were excluded due to irrelevant data [12–18]. Figure 1 demonstrated the detailed process of article identification and selection. Finally, a total of 9 articles were included [19–27]. Articles including different type of pesticides, genders and regions were considered to be independent studies. Among the 9 articles, 7 were case-control studies [19, 20, 22–24, 26, 27], and 2 were cohort ones [21, 25]. 4 researches were performed in America [21, 24, 26, 27], 2 were in Africa [19, 20], and 3 were in Europe [22, 23, 25]. 2 studies reported the correlation between risk of bladder cancer and exposure of a specific type of pesticide (herbicide [22], insecticide [26]). 3 studies adjusted for more than 3 confounding factors [19, 20, 23], and 6 studies ≤ 3 confounding factors [21, 22, 24–27]. Information was collected from interview, questionnaire or database. The quality score of each study, assessed by the Newcastle–Ottawa Quality Assessment Scale (NOS), ranged from 5 to 7 (with a mean of 6.2). Detailed characteristics of the eligible studies were shown in Table 1.

**Table 1:** Characteristics of published cohort and case–control studies on pesticide exposure and risk of bladder cancer

First Author	Published year	Study design	Period	Quality score	Region	Type of Pesticide	gender	Range of pesticide exposure	Variables of adjustment	Expossure assessment
Zahm [24]	1987	case-control	1977-1978	6	America	pesticide	male	Never vs Ever	age, sex	interview
Vecchia [22]	1990	case-control	1985-1988	5	Europe	herbicide	mixed	Never vs Ever	age, sex and smoking	questionnaire
Fincham [26]	1992	case-control	1983-1989	6	America	insecticide	mixed	Never vs Ever	age, smoking	questionnaire
Francois Viel [25]	1995	cohort	1984-1986	7	Europe	pesticide	male	Never vs Ever	age, smoking	database
Wesseling [21]	1999	cohort	1981-1993	6	America	pesticide	male/female	Never vs Ever	smoking	database
Settimi [23]	2001	case-control	1990-1992	6	Europe	pesticide	male	Never vs Ever	age, education level, marital status, smoking, alcohol consumption, diet, residence, cancer history	interview
Zarzour [19]	2008	case-control	1984-2004	6	Africa	pesticide	mixed	Never vs Ever	smoking status, marital status, education, occupation	interview
Cassidy [27]	2009	Case-control	1999-2009	7	America	pesticide	mixed	Never vs >10 years	age, gender and smoking status	interview
Amr [20]	2015	case-control	2006-2011	7	Africa	pesticide	male	Never vs Ever	education, tobacco smoke, SH infection history, environmental tobacco smoke, age, and area of residence.	database

IWED = Intensity-Weighted Lifetime Exposure Days (LED × intensity level).

LED = Lifetime Exposure Days (years of use × days per year).

### Risk assessment

**Figure 2:**
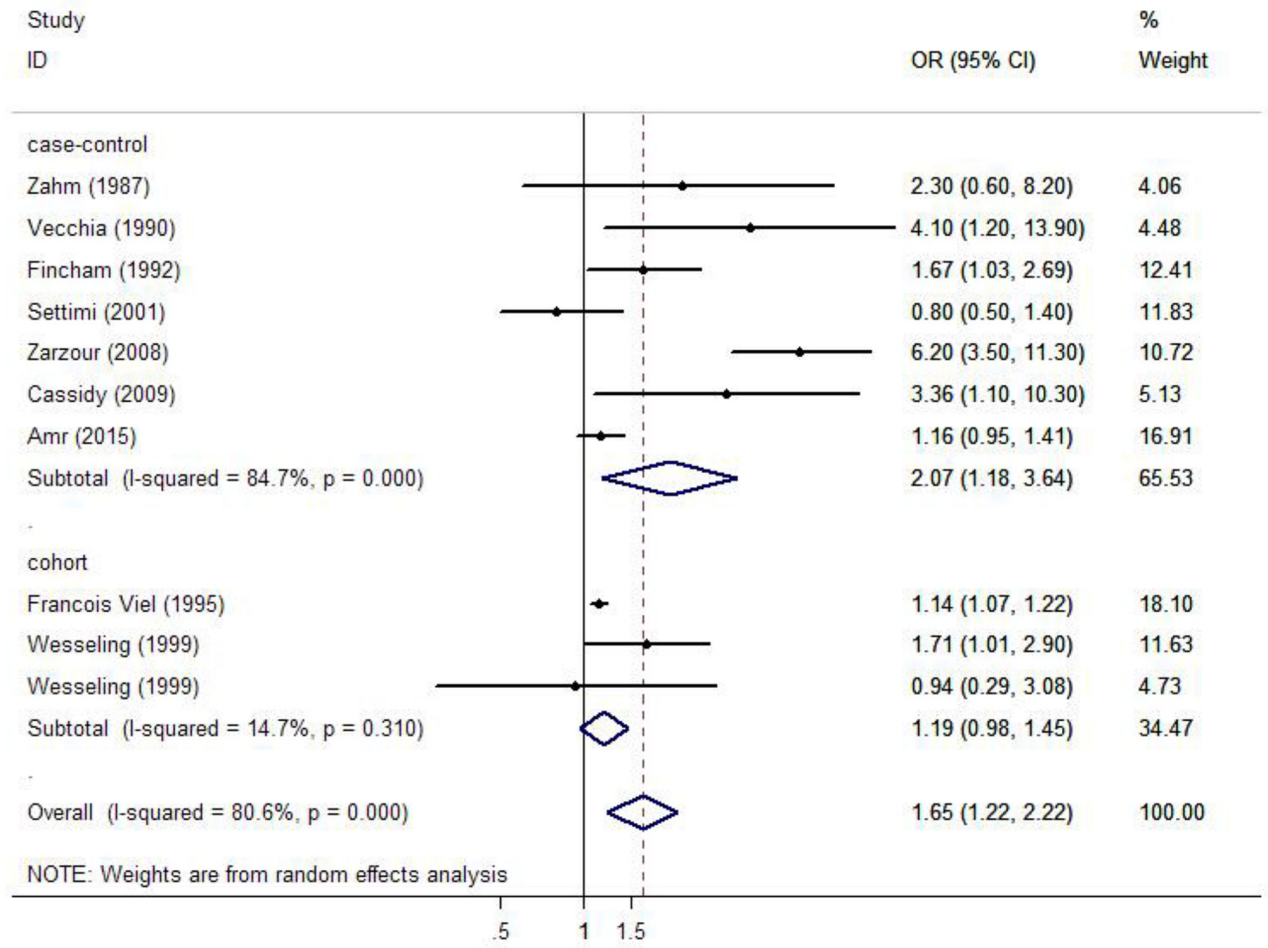
Forest plots depicting the risk estimates from included studies on the association between pesticide exposure and risk of bladder cancer

The multivariable-adjusted ORs of the highest versus lowest level of pesticide exposure, for every study and for the combination of all studies, are demonstrated in Figure 2. Among the included studies, the pooled OR estimates indicated that pesticide exposure was associated with an increased risk of bladder cancer (OR=1.649, 95% CI 1.223-2.223). Nevertheless, a statistically significant heterogeneity was also detected (I^2^=80.6%, P < 0.001). Simultaneously, we further calculated the pooled ORs grouped by design of study, and similar results were discovered in both case-control and cohort groups (OR=2.075, 95% CI 1.183-3.638, OR=1.146, 95% CI 1.074-1.223, respectively). Statistical heterogeneity cannot be avoided in the case-control subgroup (I^2^=84.7%, P < 0.001). On the contrary, statistical heterogeneity was relieved in the cohort subgroup (I^2^=14.7%, P = 0.310).

In the analysis stratified by gender, a statistically significant correlation was observed in male group (OR=1.144, 95% CI 1.076-1.217). Furthermore, when stratified by study region, we detected pesticide exposure demonstrated as a significant risk factor in bladder cancer in America (OR=1.741, 95% CI 1.270-2.388). However, no such effect was found in Europe or Africa (OR=1.187, 95% CI 0.722-1.951, OR=2.619, 95% CI 0.507-13.528, respectively). Additionally, in the subgroup analysis by exposure assessment, a significant association was observed in the database group (OR=1.148, 95% CI 1.079-1.221), but no such association was detected in neither interview nor questionnaire group (OR=2.457, 95% CI 0.755-7.989, OR=2.177, 95% CI 0.975-4.859, respectively). We also assessed whether more thoroughly adjusting for potential confounding factors affected the final result. A statistically significant correlation was observed between pesticide exposure and increased risk of bladder cancer in the group adjusted for more than 3 confounding factors (OR=1.607, 95% CI 1.065-2.423). However, no such association was observed in the group adjusted for less than or equal to 3 confounding factors (OR=1.752, 95% CI 0.659-4.657). When stratified by study quality, low-quality group illustrated that high exposure to pesticide was associated with high risk of bladder cancer (OR=1.959, 95% CI 1.081-3.550). Similar result was illustrated in high-quality group (OR=1.170, 95% CI 1.001-1.368) (Table 2).

**Table 2:** Stratified pooled odds ratio (OR) and 95% confidence intervals (CIs) for the correlation between pesticide exposure and risk of bladder cancer

Subgroup	Number of studies	OR (95% CI) Random model	OR (95% CI) Fixed model	Heterogeneity	
				*P*	*I^2^* (%)
Study design					
Case-control	7	**2.075 (1.183-3.638)**	1.395 (1.188-1.639)	0.000	84.7
Cohort	3	1.191 (0.979-1.448)	**1.146 (1.074-1.223)**	0.310	14.7
Gender					
Male	5	1.154 (1.020-1.306)	**1.144 (1.076-1.217)**	0.266	23.3
Female	1	-	-	-	-
Region					
Europe	3	**1.187 (0.722-1.951)**	1.138 (1.066-1.214)	0.050	66.7
America	5	1.741 (1.270-2.388)	**1.741 (1.270-2.388)**	0.630	0
Africa	2	**2.619 (0.507-13.528)**	1.376 (1.141-1.659)	0.000	96.5
Exposure assessment					
Questionnaire	2	**2.177 (0.975-4.859)**	1.882 (1.204-2.943)	0.181	44.2
Interview	4	**2.457 (0.755-7.989)**	2.084 (1.466-2.964)	0.000	89.0
Database	4	1.148 (1.079-1.221)	**1.148 (1.079-1.221)**	0.502	0
Control factors					
>3	3	**1.607 (1.065-2.423)**	1.479 (1.137-1.924)	0.068	48.9
≤ 3	7	**1.752 (0.659-4.657)**	1.292 (1.083-1.540)	0.000	93.7
Study quality					
High	3	**1.170 (1.001-1.368)**	1.146 (1.077-1.219)	0.166	44.3
Low	7	**1.959 (1.081-3.550)**	1.828 (1.430-2.337)	0.000	79.9

### Evaluation of heterogeneity

**Figure 3:**
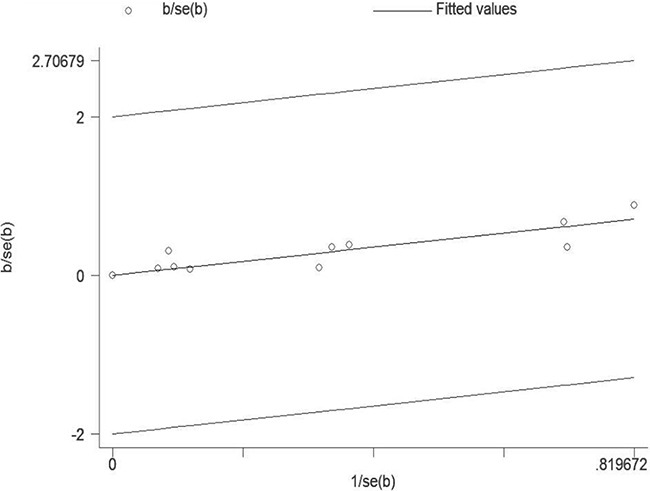
Galbraith plot analysis was used to evaluate heterogeneity It indicated that none of the included studies could be the possible source of heterogeneity.

A statistically significant heterogeneity was detected among the studies (I^2^=80.6%, P < 0.001). Therefore, the Galbraith plot test was conducted to explore the possible source of heterogeneity. However, we failed to find any of the included studies could be the possible source of heterogeneity (Figure 3).

### Cumulative meta-analysis

**Figure 4:**
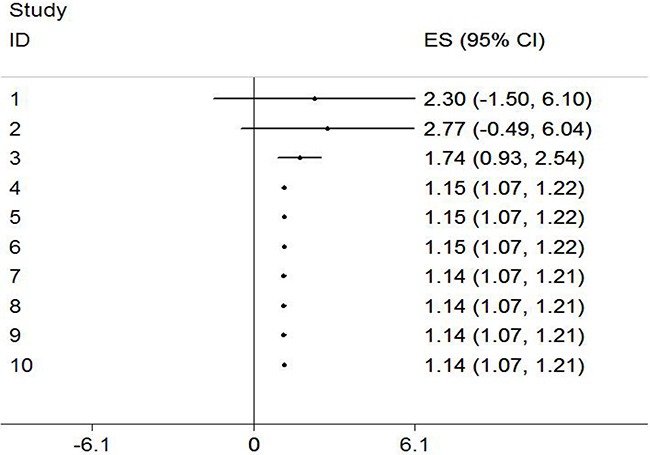
Results from cumulative meta-analysis of the relation between the pesticide exposure and risk of bladder cancer The circles and horizontal lines illustrated the accumulation of estimates as results from each study were added and the 95 % confidence intervals became narrower with the increasing sample size, implying that the accuracy of the estimates was progressively increasing by the continuous addition of studies.

Cumulative meta-analysis was carried out by ordering the studies according to publication year. The results of cumulative meta-analysis indicated that the correlation between pesticide exposure and risk of bladder cancer was in chronologic order (Figure 4). The 95 % confidence interval (95% CI) became narrower with the increase of sample size, indicating that the accuracy of the estimates was progressively increasing via the continuous addition of studies.

### Sensitivity analysis

**Figure 5:**
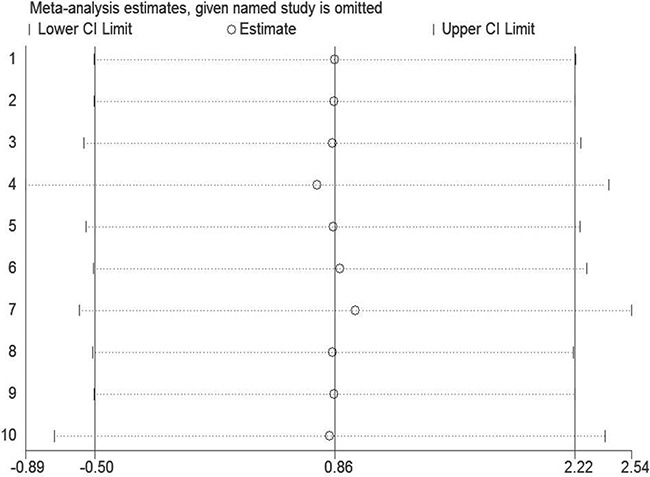
Sensitivity analysis was conducted to evaluate the effect of each study on the overall estimate by sequentially excluding one study in one turn No study could probably affect the summary of risk estimate in this study.

Sensitivity analysis was performed to assess the effect of every study on the summarized estimate by sequentially excluding one study in one turn. In our meta-analysis, we detected no study could possibly affect the pooled risk estimate (Figure 5).

### Publication bias

**Figure 6:**
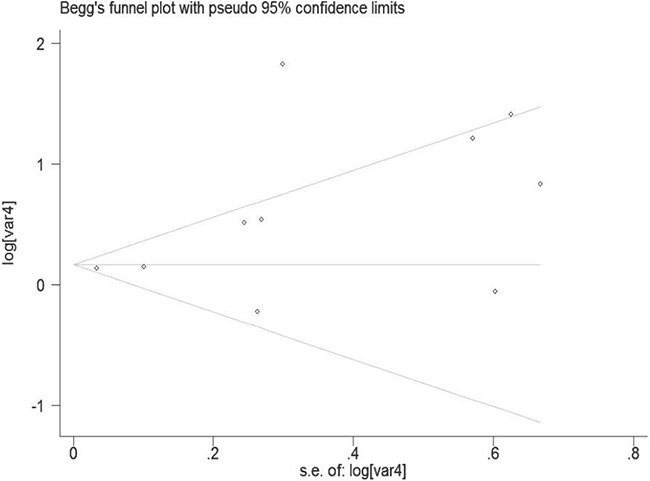
Funnel plot of the pesticide exposure and risk of bladder cancer

**Figure 7:**
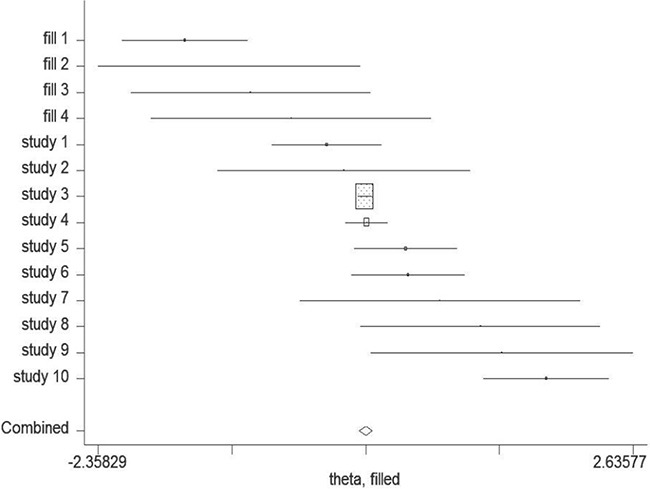
The trim-and-fill test identified 4 possible missing studies

Begg's and Egger's test was conducted to assess the possible publication bias among the including studies (Figure 6). No evidence of publication bias was detected by either way (P = 0.210, P = 0.358, respectively). The trim-and-fill test identified 4 possible missing studies (Figure 7). Nevertheless, these studies did not change the trend of our results (P = 0.310).

## DISCUSSION

Our meta-analysis summarized the results of 9 epidemiologic researches, including 7 case–control studies and 2 cohort studies. To the best of our knowledge, this is the first meta-analysis evaluating the association between pesticide exposure and the risk of bladder cancer. We found that high exposure to pesticide was related to increased risk of bladder cancer. We used quantified Q test and I^2^ test to evaluate the degree of heterogeneity among the eligible studies. A statistically significant heterogeneity was discovered among the overall studies (I^2^=80.6%, P < 0.001). Therefore, we further conducted the Galbraith plot test to assess the possible source of heterogeneity. However, we failed to find any of the included studies could be the possible source of heterogeneity.

To identify the possible source of heterogeneity, subgroup analysis was conducted. We found that pesticide exposure was associated with an increased risk of bladder cancer in both case-control and cohort groups (OR=2.075, 95% CI 1.183-3.638, OR=1.146, 95% CI 1.074-1.223, respectively). Heterogeneity could be avoided in the cohort subgroup (I^2^=14.7%, P = 0.310). Based on the characteristics of case-control and cohort study, cohort study is a better method to elucidate the relationship between pesticides exposure and risk of bladder cancer, and the result from cohort study is more reliable since case-control study has more confounding factors, which might be a possible source of heterogeneity. Furthermore, we performed a subgroup analysis by exposure assessment, and a significant association was observed in the database group (OR=1.148, 95% CI 1.079-1.221), but no such association was detected in neither interview nor questionnaire group (OR=2.457, 95% CI 0.755-7.989, OR=2.177, 95% CI 0.975-4.859, respectively). Furthermore, we found no statistically significant heterogeneity in the database group (I^2^=0.0%, P = 0.502). On the contrary, moderate heterogeneity was detected in the self-administered questionnaire group (I^2^=44.2%, P = 0.181), which suggested systematic recorded database for data collection should be a better choice in the future studies which could avoid potential heterogeneity. Additionally, we considered the quality of study and number of confounding factors could be a potential source of heterogeneity. These results indicated that the heterogeneity of the included studies could have resulted in an exaggeration of the risk estimate.

Furthermore, when stratified by study region, we detected pesticide exposure demonstrated as a significant risk factor in bladder cancer in America (OR=1.741, 95% CI 1.270-2.388). However, no such effect was found in Europe or Africa (OR=1.187, 95% CI 0.722-1.951, OR=2.619, 95% CI 0.507-13.528, respectively). As we mentioned before, only 2 studies and 3 studies concerned the relationship between pesticide exposure and risk of bladder cancer in Africa and Europe, respectively. We do admit that small number of studies included in the meta-analysis, which may have a negative impact on the conclusion of this study. Therefore, the results in our analysis were considered to be preliminary results and the conclusion should be treated with caution. More multi-center, large-sample and well-designed studies are of great necessity to better illuminate the relationship between pesticide exposure and risk of bladder cancer in different regions in the future.

When we restricted to studies adjusted for more than 3 confounding factors, we found that the association was more robust (OR=1.607, 95% CI 1.065-2.423) than that reported in the overall analysis, which indicated that the association may have been diluted by poor study methodologies. This is consistent with the result of studies judged by NOS score. So pesticides exposure is possibly an independent risk factor for bladder cancer.

The biological mechanisms underlying the correlation between pesticides exposure and the carcinogenesis of bladder cancer still remains unknown. Nevertheless, several potential mechanisms could be conceivable. Exposure to pesticides might bring about over-expression of reactive oxygen species (ROS), which was sufficient to disorder antioxidant defense mechanisms and result in extensive DNA damage and protein damage [28]. Additionally, pesticides could bind to and displace endogenous ligands of steroid nuclear receptors, including androgen and estrogen receptors, subsequently aberrantly activating receptor function and leading to changes in gene expression network [29]. Previous research illustrated that trivalent pesticide related chemical could induce protein carbonylation and oxidative DNA damage in human urothelial cells, and finally result in bladder cancer [30]. However, more researches are still needed to elucidate the possible biological mechanisms.

Our study also has several limitations. Firstly, although no publication bias was detected in our study by either Begg's or Egger's test, the selection strategy of published studies in English and Chinese merely and exclusion of study without sufficient information could lead to potential publication bias. Moreover, our searching was restricted to published articles, which could also cause possible bias to affect our ultimate findings. Secondly, both cohort and case-control studies were recruited in our study. Considering the existing heterogeneity, it might be inappropriate to select a single global effect estimate to summarize the data, and the pooled estimates in our study should be treated with caution. Therefore, we conducted subgroup analysis to explain the possible sources of heterogeneity. Additionally, half of the studies in our analysis were case-control studies, which could possibly cause selection and recall bias. Thirdly, a meta-analysis cannot solve a problem with confounding factors that could be internal in the recruited studies. Insufficient control of known confounding factors could bring about bias in direction either toward exaggeration or underestimation of the risk estimates [31]. In our study, the possibly insufficient control of confounding factors seemed to be a particular concern in the studies included: only 3 studies adjusted for three or more than three control factors. Many studies failed to adjust for other pesticides most highly correlated with exposure one. Therefore, potential or unknown confounding factors could not be completely excluded in the results of our meta-analysis. Finally, we also tried to conduct a dose–response analysis to demonstrate the correlation between exposure level of pesticide and risk of bladder cancer. However, the included studies failed to provide the exact number of cases and controls in each exposure category. Therefore, a dose–response analysis was unable to be carried out. Furthermore, a wide range of values for the cutoff points for the lowest and highest level of the pesticide exposure was observed in the included studies, which could possibly impact the result of this meta-analysis.

In conclusion, our meta-analysis indicated that pesticide exposure was associated with an increased risk of bladder cancer. Further researches should be conducted to confirm the findings in our study and better clarify the potential biological mechanisms.

## MATERIALS AND METHODS

### Literature search

In order to get a general view on the correlation between pesticide exposure and the risk of bladder cancer, a systematic and comprehensive searching strategy was conducted. We searched for the publications updated to February 2015 using Pubmed, Web of Science, Cochrane Library, and the Chinese National Knowledge Infrastructure (CNKI) databases. (Bladder cancer) AND (pesticides OR herbicides OR fungicides OR insecticides) were selected as keywords to identify the publications. We evaluated all the potentially relevant publications via checking both their titles and abstracts, and articles meeting the eligible criteria were retrieved. Additionally, other relevant articles were retrieved either by evaluating the cited references in the recruited publications or reviews regarding the association between pesticide exposure and the risk of bladder cancer. The current study was planned, performed, and illustrated in accordance with the standards of quality for meta-analysis [32].

### Including and excluding criteria

Each included article was assessed whether the following criteria were met: (1) case-control or cohort study evaluating the potential relationship between pesticide exposure and the risk of bladder cancer; (2) exact data in both case and control groups ought to be identified; (3) articles published before February 2015 written in either English or Chinese; (4) results including relative risk (RR) or odds ratio (OR) and its 95% confidence intervals (95% CIs), or providing us with sufficient data to calculate them. If more than one publication from the same population were obtained, the latest study was eligible for inclusion. Studies with overlapping or insufficient data were excluded.

### Data extraction

We extracted data from the recruited articles including name of the first author, publication year, country, design of study, sample size, exposure assessment, adjusted effect estimates for all categories of pesticide exposure, and matched or adjusted variables in the analysis. Considering bladder cancer is a relatively rare disease, RR was considered the same as OR. Therefore, we chose OR as the result to evaluate the potential correlation between pesticide exposure and bladder cancer risk. Two investigators independently extracted data from all the potential publications in case of mistakes and omissions. We chose group discussion and consulted a third reviewer to resolve any discrepancy.

### Quality assessment

The quality of each article was assessed using the Newcastle–Ottawa Quality Assessment Scale (NOS) (http://www.ohri.ca/programs/clinical_epidemiology/oxford.asp) by the same two authors. Any disagreement was discussed via a re-evaluation of the original study by a third reviewer. NOS is an eight-item tool which allows for the evaluation of the population selection, study comparability, and exposures for both cohort and case-control studies. Analysis of the scale is conducted by awarding ‘stars’ for high-quality elements. The number of the stars is counted and used to assess the quality of the study in a quantitative way. The scale of the scores is 0–9. We considered scores of <7 and ≥7 as low and high quality studies, respectively.

### Statistical analysis

We used OR and 95% CI to assess the strength of the correlation between pesticide exposure and the risk of bladder cancer. Both the Fixed-effect model with the method of Mantel-Haenszel [33], and the random-effect model with the method of DerSiomonian and Laird were chose to supply pooled estimation of the relationship between pesticide exposure and the risk of bladder cancer [34]. The subgroup analysis was conducted by study design, study region, quality of study, exposure assessment, and the number of control factors.

We also use quantified Q test [34] and I^2^ [35] test to assess the degree of heterogeneity among the eligible studies. Heterogeneity was identified with a significance level of P < 0.10. The value of I^2^ was selected to evaluate the extent of heterogeneity (no heterogeneity I^2^ < 25%; moderate heterogeneity I^2^ = 25–50%; large heterogeneity I^2^ > 50%). When I^2^ < 25%, results of fixed-effect model were chose, otherwise, results of random-effect model were chose. Furthermore, the Galbraith plot test was performed to explore the possible source of heterogeneity [36], and if necessary, a second analysis was carried out after excluding the studies which is possibly causing heterogeneity.

We also conducted the cumulative meta-analysis by ordering the studies according to the publication year. Sensitivity analysis was performed to evaluate the effect of each study on the overall estimate.

Publication bias was assessed by Egger's [37] and Begg's test [38]. The statistically significant level was set at 0.05. We also selected the trim-and-fill test to evaluate the potential publication bias [39]. The trim-and-fill method suggests that the effect sizes of all the studies distribute normally around the central point. If asymmetry is discovered, it adjusts for the potential effects that unpublished studies could have on the measured result.

All statistical analyses were performed via using STATA version 11 (StataCorp, College Station, Texas, USA).
